# Can Dental Office Lighting Intensity Conditions Influence the Accuracy of Intraoral Scanning?

**DOI:** 10.1155/2021/9980590

**Published:** 2021-05-27

**Authors:** Anca Jivanescu, Andrei-Bogdan Faur, Raul Nicolae Rotar

**Affiliations:** ^1^Department of Prosthodontics, University of Medicine and Pharmacy “Victor Babes”, TADERP Research Center, Timisoara, B-dul Revolutiei 1989, No 9, 300580, Romania; ^2^Victor Babes University of Medicine and Pharmacy of Timisoara (UMFT), Romania

## Abstract

The aim of this study was to evaluate the influence of different settings of ambient light intensity inside the dental office on the accuracy (trueness and precision) of an intraoral scanner (IOS). A full crown preparation was conducted on a resin molar which was scanned using a high resolution extraoral scanner to obtain a reference model. Six light settings were chosen based on the most clinically relevant light conditions inside the workspace, and the preparation was scanned using an intraoral scanner (PlanScan, Planmeca). The obtained data was analyzed using a professional 3D quality control software (Geomagic Control X). There was no statistically relevant difference between the groups when regarding trueness, although a slight influence of the light intensity could be observed on the trueness values. Regarding precision, the best results were obtained in the 3800 lux group, with the other groups presenting close values, excepting the extreme values (400 lux and 11 000 lux) groups that proved to be the most deficient.

## 1. Introduction

The development of technology applicability in dentistry and the trend towards digitalization with regard to all the potential benefits that come with it have brought great improvements in the intraoral scanners (IOS), making them more accurate and reliable than ever before [1–5]. The improvements of the intraoral scanners allow obtaining digital impressions with marginal gaps within the clinically acceptable range for various types of restorations and clinical situations [6], considerably decreasing the time needed for impression making, while undeniably improving the comfort of the patient [7–11].

The accuracy of an intraoral scanner can be influenced by many factors including the hardware and software technology of the intraoral scanner [12–14]; the scanning protocol [15–18]; the calibration process of the IOS [14]; the scanned surface traits like texture, height, and geometry [19]; and the light intensity and color temperature of the ambient lighting conditions [20–25]. Most of these factors cannot be manipulated in any way by the clinician; however, the ambient lighting conditions can be easily modified to certain parameters only for the scanning stage, with no cost and insignificant time consumption. Multiple individual ambient lights in the working space, dimmable light switches, and dental chair lights with multiple intensity levels allow the clinician to increase or decrease the light intensity that reaches the scanned surface during the digital scanning process therefore influencing the conditions of the scan. Some clinical studies confirmed that the ambient lighting has a significant influence over the accuracy of an intraoral scanner [22, 23].

Two aspects are to be taken into consideration when comparing the accuracy of a digital impression: trueness and precision [26, 27]. These two independent variables when analyzed can provide a certain overview on the accuracy. Trueness refers to how detailed and close to reality is the digital impression, and it changes under different settings. Precision refers to how similar are repeated digital impressions taken under the same conditions, therefore the degree of reproducibility [28–32]. The ideal ambient lighting condition should enable the IOS to produce the highest trueness and precision.

There are strict standards and recommendations for the lighting conditions inside the dental office provided by the European Committee for Standardization in BSI Standards Publication Light and lighting (EN 12464-1 : 2011) [33] as well as different scientific studies on the matter. Between 500 and 1000 lux-illuminance is recommended for the examination room while up to 10 000 lux-illumination is recommended for the operating area [34, 35]. The light intensity varies according to the number of ambient lights on the celling, the active lighting level of the dental chair light, and the natural light that is able to enter the room which is constantly influenced by the window setting, the weather, and the time of the day. Analyzing the differences in digital impression accuracy obtained with different ambient light settings in the dental office could help to clarify the issue regarding how much does the lighting conditions influence the intraoral scanning accuracy.

The aim of this study is to evaluate the influence of different settings of ambient light inside the dental office working space on the accuracy of an intraoral scanner and to quantify the differences in accuracy over the different settings.

## 2. Material and Methods

**Figure 1:**
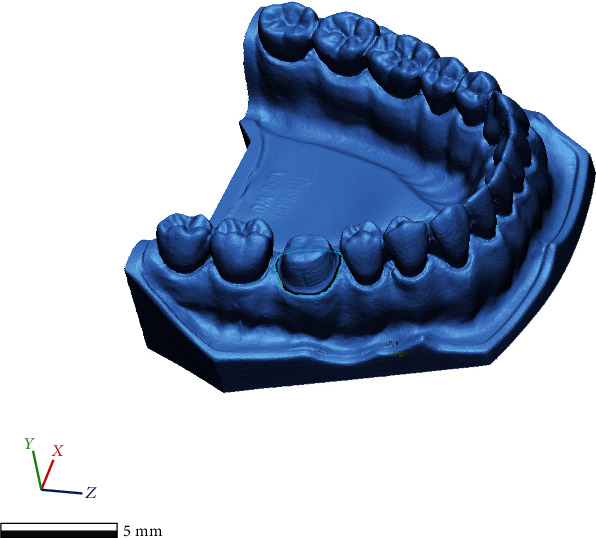
The reference mesh obtained at the high resolution extraoral scanner (D700 3D scanner, 3Shape).

The study was conducted in the Prosthodontics Clinic, using a typodont (AG-3; Frasaco) inserted in the mandibular articulation of a dental mannequin (Phantom head PK-2 TSE; Frasaco). A full crown preparation with deep chamfer margin was conducted on the right mandibular first molar of the typodont. The typodont was sent to a dental laboratory in order to be scanned with a high resolution extraoral scanner (D700 3D scanner, 3Shape). The calibration of the extraoral scanner was performed according to the manufacturer's instructions in order to obtain a gold standard scanning. By opening the “3Shape ScanServer” software with the scanner being connected to the desktop computer, the assisted calibration process was initialized. The calibration object, provided by the manufacturer inside the calibration kit, was inserted inside the scanner, and the calibration process was started from within the software. During this process that takes up to 3 minutes, the scanner runs through its predefined motions calibrating itself. After the calibration was successful, the calibration object was removed, and the scanner was ready for use. The scanner manufacturer claims a high accuracy < 20 microns for this scanner. Therefore, the typodont was digitized in order to obtain a standard tessellation language (STL) file which would serve as the reference scan (Figure 1).

**Figure 2:**
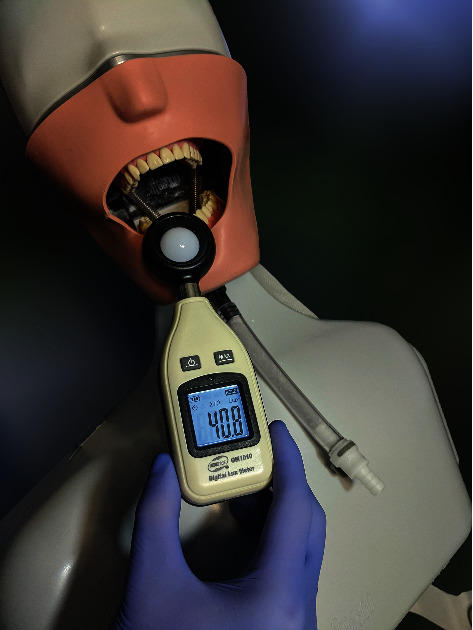
The lux meter GM1010 measuring the light intensity in the area of the typodont.

Inside the dental office, a digital lux meter (GM1010; Benetech) with the measuring range of 0 ~ 200 000 lux was used to measure and quantify the light intensity under specific conditions that would be most relevant and plausible: neon ambient light on a cloudy day, measuring 400 lux; neon ambient light on a sunny day, measuring 1000 lux; chair light at half intensity, measuring 3300 lux; chair light at half intensity and neon ambient light, measuring 3800 lux; chair light at full intensity, measuring 10 000 lux; and chair light at full intensity and neon ambient light, measuring 11 000 lux. The measurement was done placing the diode of the lux meter in the same spot as the typodont (the typodont was fixed to reassure that it will not be accidentally moved during the entire duration of the scans) (Figure 2).

Six groups were therefore created based on the six light intensity settings: group 1 = 400 lux, group 2 = 1000 lux, group 3 = 3300 lux, group 4 = 3800 lux, group 5 = 10 000 lux, and group 6 = 11 000 lux.

**Figure 3:**
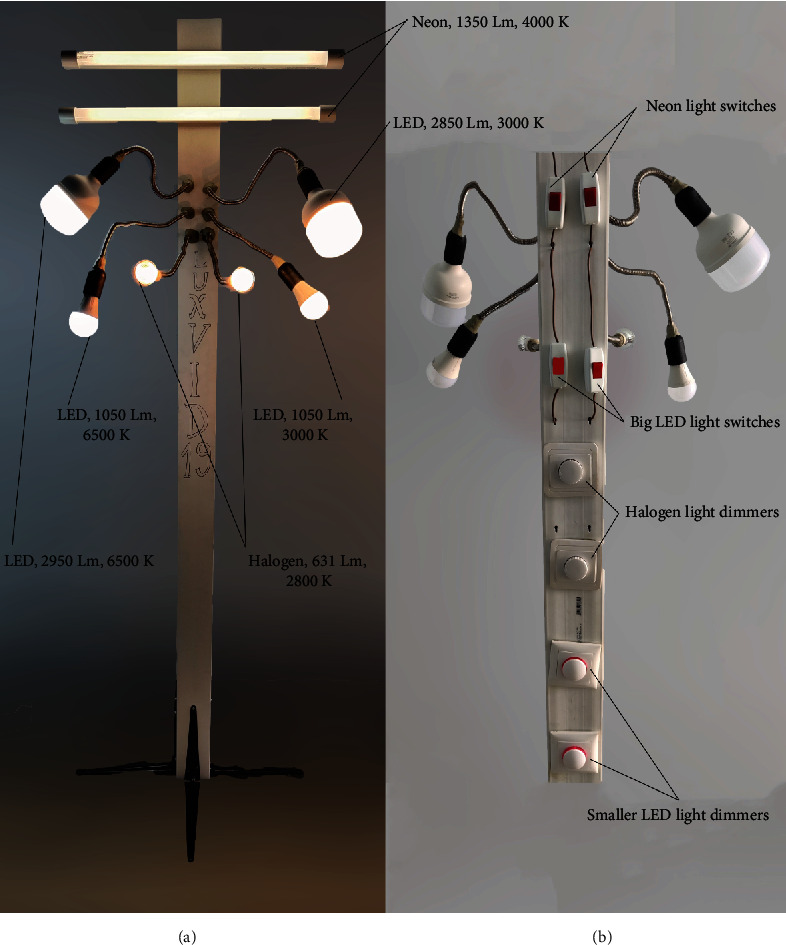
Lighting device named “Luxvid 19” designed specifically to aid in this study: (a) front view; (b) back panel containing the switches and dimmers.

A device was purposefully designed and manufactured to accurately offer precise and reproducible lighting conditions that were checked using the lux meter. Eight light sources were connected in a parallel circuit, so that they could function individually or together, depending on the clinician's desire. The entire circuit with the light sources was encapsulated inside a long rigid plastic case with stands at the bottom to sustain the weight of the entire device. The device would be connected to a 220–240 V socket via a standard AC power plug. In exact order from the top to the bottom, the light sources of the device consist of two separate fixed neon tubes (G13, Kingfisher, 1350Lm, 4000K, 18W) each activated by an individual on and off switch, one big light-emitting diode (LED) light bulb (T100, Lohuis, 2950Lm, 6500K, 30W) inserted inside a flexible aluminum tube that would allow mobility and positioning options and activated by an on and off switch, another big LED light bulb (T100, Hepol, 2850Lm, 3000K, 30W) inserted inside a flexible aluminum tube and activated by an on and off switch, one smaller but dimmable LED light bulb (BE27-12-DIM-CW, Hoff, 1050Lm, 6500K, 12W) inserted inside a flexible aluminum tube and activated by a dimmable switch that allows different light intensity output, another smaller and dimmable LED light bulb (BE27-12-DIM-WW, Hoff, 1050Lm, 3000K, 12W) inserted inside a flexible aluminum tube and activated by a dimmable switch, and two final halogen lamps (TG-2205.0150, Total Green, 631Lm, 2800K, 50W) each inserted inside a flexible aluminum tube and activated by a separate dimmable switch (Figure 3).

Due to the multitude of light sources, dimming option, and parallel circuit functionality, this device was able to provide the desired light intensity in an accurate and reproducible manner. For the subsequent intraoral scans, this device was the only one used to provide the exact desired light intensity, switching from one chosen light setting to the other. The other light sources in the dental office were turned off, and the light intensity was measured with the lux meter for reassurance prior to each scan.

**Figure 4:**
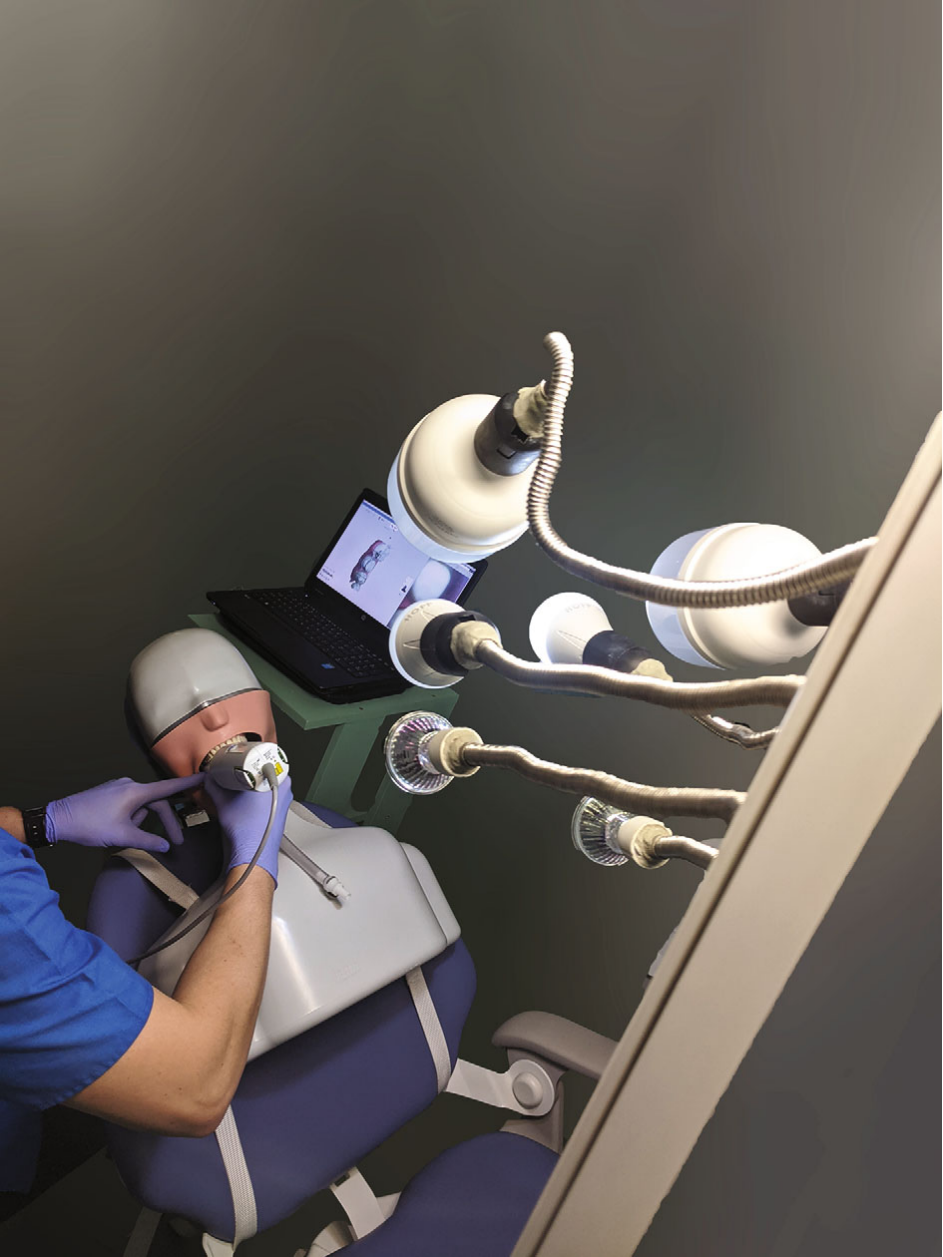
Settings of the scanning protocol using the Planmeca PlanScan IOS.

The typodont preparation was scanned using an intraoral scanner (Planmeca PlanScan) for 5 times under each chosen lighting setting therefore obtaining 6 groups, each containing 5 STL files. The scans were conducted by the same experienced prosthodontist, following the scanning protocol of the IOS manufacturer, the typodont and mannequin being mounted and secured on the dental chair, and the lighting device being set at a fixed distance from the typodont (Figure 4).

The obtained STL files were methodically organized and stored (Table 1).

**Figure 5:**
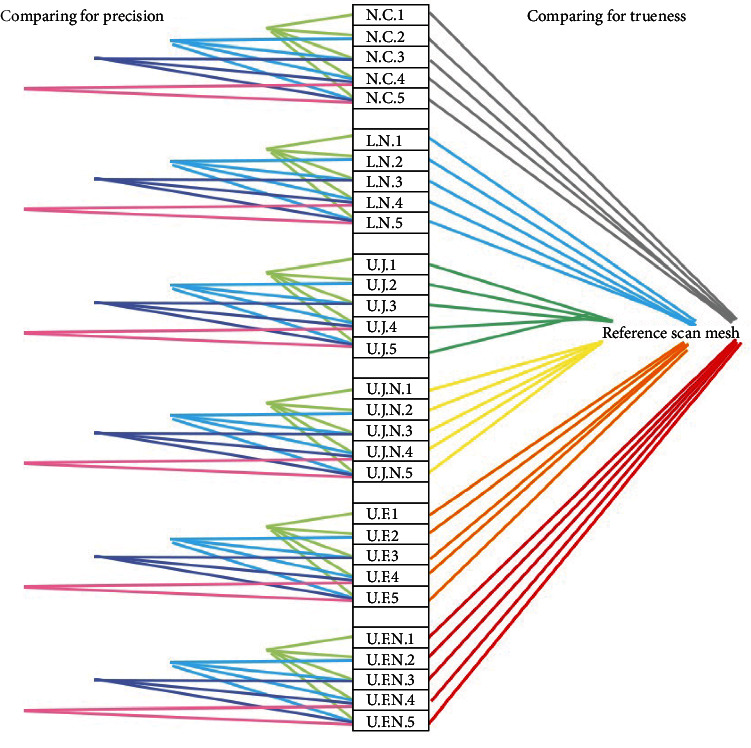
Diagram illustrating mesh comparison order to obtain trueness values and precision values. The center column displays the names of the STL files and subsequent meshes that were analyzed. The colored lines suggest the comparisons executed between the meshes. In order to obtain the numerical data for trueness, each mesh was compared with the reference scan mesh (as shown in the right section of the figure). In order to obtain the numerical data for precision, each mesh was compared within its group of similar light intensity (as shown in the left section of the figure).

An inspection and metrology software (Geomagic Control X) was used to assess the scanned data. Geomagic Control X is a complete metrology grade, quality control software that is equipped with powerful tools designed to improve multiple existing workflows. It provides a full range of user-friendly, intuitive controls, alongside traceable, repeatable workflows for a more efficient quality measurement process. The software has multiple accurate functions such as “3D Compare” that achieve highly accurate measurements, and its sophisticated CAD-based dimensioning tools allow industry professionals to quickly conduct various analyses. The software also supports STL files, the encoding format of our data obtained from the scanner. To obtain the data that reflects trueness, the meshes of the IOS scans were compared to the mesh of the reference scan (Figure 5).

**Figure 6:**
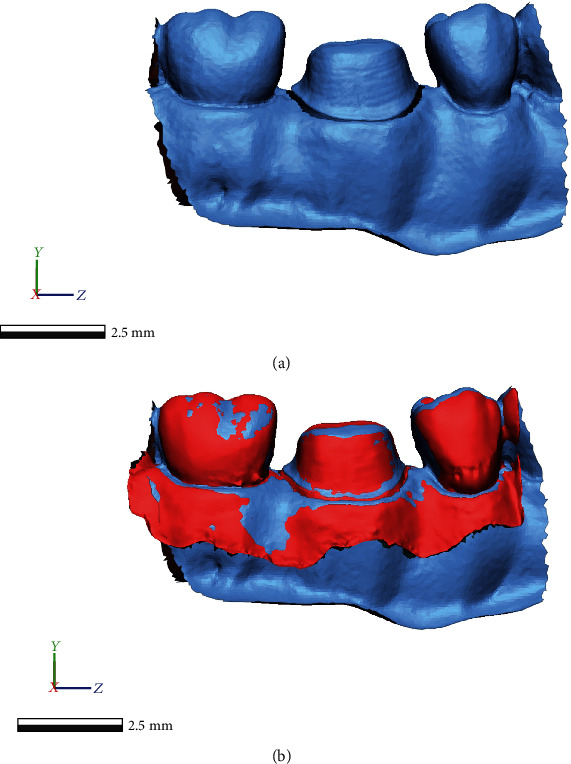
Trimmed reference mesh (a) and the superimposing of an IOS mesh (b).

The reference data was uploaded into the software and was trimmed to the area of interest including the prepared tooth and one mesial and distal tooth in order to match the IOS scanned area consequently facilitating the superimposing of the two meshes. Only the prepared tooth surface was analyzed between the meshes; therefore, the preparation area of the lower first molar was carefully isolated on the reference mesh (Figure 6).

**Figure 7:**
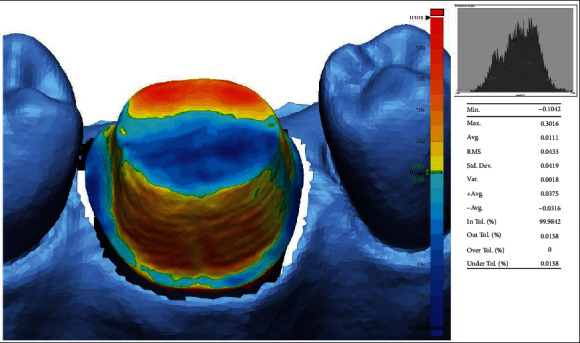
Color-coded map of the prepared tooth surface displaying deviations between ±100 *μ*m.

An “initial alignment” was executed to superimpose the IOS mesh over the reference mesh followed by the “best fit alignment” to secure a precise overlapping. The “3D Compare” function was used to analyze the deviation between the reference and measured data by projecting all paired points onto the reference data. A color-coded map was rendered displaying the deviation patterns of the investigated surfaces. The color-coded map was set to display deviations between ±100 *μ*m (Figure 7).

Outward displacement is displayed towards the red spectrum while inward displacement is displayed towards the blue one. Green suggests no deviation between the analyzed mesh and the reference one. The procedure was executed for all 30 IOS meshes in order to obtain the mean and standard deviation of each mesh consequently obtaining the trueness values.

To obtain the precision values, each IOS mesh was compared with all the other meshes within its group of similar lighting conditions. The entire alignment process was repeated for each 3D comparison; the color-coded map was rendered, and the standard deviation alongside other relevant numerical data was obtained so that the precision of each group could be calculated.

The standard deviation data obtained from the metrology software was uploaded into a statistical software (MedCalc) in order to conduct the statistical analysis. The Kolmogorov Smirnov test was conducted to evaluate if the values were normally distributed. To further analyze the data, Kruskal-Wallis test was used on the nonparametric data set while One-Way ANOVA was used on the parametric data set. The *p* value for level of significance was set to *p* < 0.05. The assumed null hypothesis was that there would be no significant difference in accuracy between the scans obtained under the selected lighting conditions.

## 3. Results

The trueness values are presented in Table 2 while the precision values are presented in Table 3.

**Figure 8:**
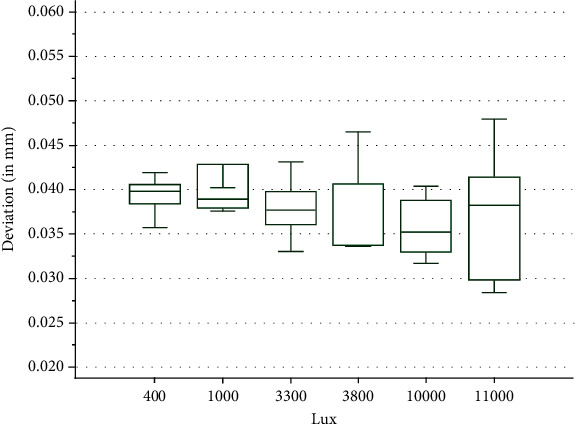
Boxplot of the trueness values.

By conducting the Kolmogorov Smirnov test on the trueness values, it resulted that only part of the data was normally distributed. By conducting the Kruskal-Wallis test on the trueness values, it was observed that the U.F. group (10 000lux) displayed the best level of trueness to the reference data, the deviation being the lowest of all the groups with a median of 0.0352 mm. In descending order regarding the trueness, the other groups were ranked as follows: U.F.N group (11 000 lux) with a slight decrease in trueness, U.J.N. group (3800 lux) closely followed by U.J. group (3300 lux), L.N. group (1000 lux) with a severe decrease in trueness, and lastly N.C. group (400 lux) displaying the lowest level of trueness out of all the groups. This suggests an increase in trueness with the increase of light intensity from 400 lux to 10 000 lux where the trueness starts to diminish if the light is increased after this threshold (Figure 8). Despite the differences observed between the groups, the Kruskal-Wallis test returned a value of *p* = 0.53, failing to reject the null hypothesis. Consequently, the test indicated that there is no statistically relevant difference between the groups.

**Figure 9:**
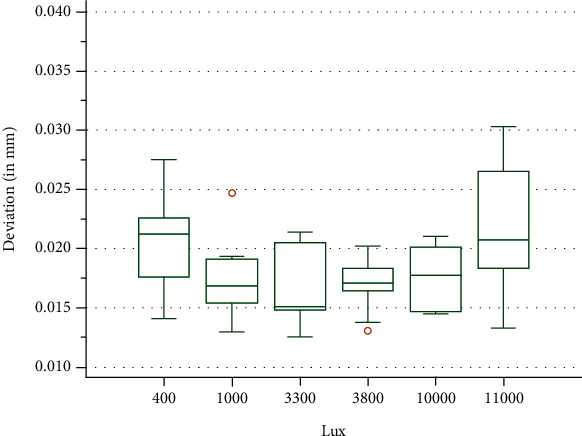
Boxplot of the precision values.

The Kolmogorov Smirnov test applied on the precision values revealed that the data were normally distributed. In terms of precision, the best results are obtained in the U.J. group (3300 lux) closely followed with almost similar values by the U.J.N group (3800 lux) and L.N. group (1000 lux). The N.C. group (400 lux) was even more unsatisfactory, and the U.F.N group (11000 lux) proved to be the most deficient when considering precision (Figure 9). The One-Way ANOVA test returned a value of *p* = 0.016 rejecting the null hypothesis and showing that at least two groups differ significantly.

## 4. Discussion

The accuracy of an intraoral scan can be influenced by a number of external factors such as the geometry of a preparation, humidity levels, or the operator's experience [36]. However, there is another potentially important influencing factor related with the ambient light condition.

A number of studies evaluated the impact of different illumination sources on the accuracy of digital scans.

Voisin et al. [37] investigated the influence of external light on 3D scanners, and they concluded that ambient light can generate errors in the final scan.

Another study conducted by Toshio Arakida et. al [20] observed the influence of ambient light on the accuracy and speed of scanning and concluded that under the condition of 500 lux and 3900 K; the trueness of the digital impression was highest among the test groups, and regardless of the color temperature, the time needed for the digital impression was longer at 2500 lux than at 0 lux or 500 lux.

Revilla-León et al. [21] have concluded that the dental chair light should be avoided during the intraoral scanning phase and that around a 1000 lux-lighting condition is required to maximize the accuracy of the tested IOS.

Wesemann et al. [38] concluded that the influence of the ambient light varies greatly depending on the utilized intraoral scanner. Furthermore, it is stated that only full arch scans proved to be significantly influenced and that there was no clinically relevant effect on 4-unit scans.

To our strength, the present study evaluated clinically relevant light intensity values that are common inside most of the dental offices and that could be easily obtained without any additional lighting devices. When deciding on the light intensity levels that were about to be tested in the study, only the ones that could be produced and easily changed by the common lighting devices inside the operating area were selected. To eliminate error and light fluctuation, the special lighting device described in the study was constructed in order to produce the exact desired light intensity that is also constant and reproducible. Despite being an *in vitro* study, we tried to simulate some of the *in vivo* conditions, the entire experiment being constructed around the idea of being as clinically relevant as possible. The typodont was placed inside a mannequin that had acrylic cheeks that could arguably restrict the light reaching the scanned area during the scanning process alongside the hand of the clinician holding the IOS, especially on the posterior areas. But after all, there is little ambient light reaching the scanned area when scanning inside the mouth of a real patient, so it is expected that the ambient light could only have limited influence over the scanning process.

The statistical data analysis of the experimental data revealed that ambient light intensity has little overall influence over the accuracy of the intraoral scanner. The variation of the trueness values between the groups of different light intensity tested must be attributed to other factors as the trueness values did not change in correlation with the increase or decrease of the light intensity. This was also seen in the Krusal-Wallis rank-based significance test that failed to reject the null hypothesis, stating that there were no significant differences found between the data when regarding trueness. The analysis of the precision values presented notable differences, the One-Way ANOVA test rejecting the null hypothesis and stating that there are statistically significant differences. The best values for precision were obtained around the U.J. (3300 lux) and U.J.N. (3800 lux) groups, with a notable decrease in precision being seen at lower light intensity in the N.C. group (400 lux) or significantly higher light intensity in the U.F.N group (11000 lux). Nonetheless, the inconsistency between the two accuracy factors furthermore suggests that the results are inconclusive. For the conditions tested in the current study and regarding the intraoral scanner utilized, there seems to be little influence of the ambient light on the accuracy of the scans.

Furthermore, our study has a number of limitations. Being an *in vitro* study, it does not take into consideration other influencing factors such as saliva, blood, or movement of the patient. Additional research and studies must be conducted to fully comprehend the influence of lighting conditions on the accuracy of the existing intraoral scanners.

## 5. Conclusions

Despite some differences being observed between the trueness and precision data obtained under the different light intensities, they were not clinically relevant to conclude a considerable influence of the ambient light on the accuracy of the intraoral scanning. Therefore, a specific ambient light setting in the dental office for the scanning protocol cannot be recommended. Overall, there could not be attributed any statistical significance in the accuracy of the intraoral scanner used, under the different lighting conditions that were simulated in this test.

Clinically speaking, the fact that there is little influence of the ambient light conditions over the scanning accuracy comes as a reassuring advantage for the clinician as there is less room for external error when considering the use of digital impressions.

## Figures and Tables

**Table 1:** Description of data organization, light intensity, and evaluated conditions.

Group #	Lux	Simulated conditions	Scan #
Group 1–N.C.	400	Neon ambient light on a cloudy day	N.C.1
			N.C.2
			N.C.3
			N.C.4
			N.C.5
Group 2–L.N.	1 000	Neon ambient light on a sunny day	L.N.1
			L.N.2
			L.N.3
			L.N.4
			L.N.5
Group 3–U.J.	3 300	Chair light at half intensity	U.J.1
			U.J.2
			U.J.3
			U.J.4
			U.J.5
Group 4–U.J.N.	3 800	Chair light at half intensity and neon ambient light	U.J.N.1
			U.J.N.2
			U.J.N.3
			U.J.N.4
			U.J.N.5
Group 5–U.F.	10 000	Chair light at full intensity	U.F.1
			U.F.2
			U.F.3
			U.F.4
			U.F.5
Group 6–U.F.N.	11 000	Chair light at full intensity and neon ambient light	U.F.N.1
			U.F.N.2
			U.F.N.3
			U.F.N.4
			U.F.N.5

**Table 2:** Data obtained when comparing for trueness.

Scan #	Lux	Std. Dev.	Min.	Max.	Avg.
N.C.1	400	0.0419	-0.1042	0.3016	0.0111
N.C.2	400	0.0357	-0.0859	0.2964	0.0133
N.C.3	400	0.0398	-0.1279	0.3204	0.0149
N.C.4	400	0.0393	-0.134	0.2805	0.0113
N.C.5	400	0.0401	-0.1344	0.2592	0.0149
L.N.1	1000	0.0508	-1.4853	0.2996	0.0144
L.N.2	1000	0.0376	-0.1489	0.2378	0.0111
L.N.3	1000	0.038	-0.1193	0.257	0.013
L.N.4	1000	0.0389	-0.1337	0.2262	0.0159
L.N.5	1000	0.0402	-0.1071	0.2792	0.0181
U.J.1	3300	0.0371	-0.1145	0.2422	0.0128
U.J.2	3300	0.0387	-0.0945	0.2474	0.0197
U.J.3	3300	0.0431	-0.135	0.2526	0.0191
U.J.4	3300	0.0377	-0.0925	0.2468	0.0173
U.J.5	3300	0.033	-0.0951	0.2522	0.0117
U.J.N.1	3800	0.0465	-0.1461	0.2364	0.021
U.J.N.2	3800	0.0336	-0.089	0.3087	0.0132
U.J.N.3	3800	0.0337	-0.0987	0.2396	0.0118
U.J.N.4	3800	0.0379	-0.0839	0.2393	0.0161
U.J.N.5	3800	0.0387	-0.1117	0.2435	0.015
U.F.1	10000	0.0317	-0.0924	0.2388	0.012
U.F.2	10000	0.0383	-0.0908	0.2368	0.0141
U.F.3	10000	0.0352	-0.0887	0.2252	0.0169
U.F.4	10000	0.0403	-0.1073	0.2517	0.0166
U.F.5	10000	0.0334	-0.1077	0.2428	0.0094
U.F.N.1	11000	0.0479	-0.1066	0.2286	0.0231
U.F.N.2	11000	0.0303	-0.0792	0.2297	0.0091
U.F.N.3	11000	0.0284	-0.0773	0.2354	0.0092
U.F.N.4	11000	0.0382	-0.0994	0.2459	0.0154
U.F.N.5	11000	0.0392	-1.3395	0.2524	0.0175

Scan #: the name of the STL file and subsequent mesh that was analyzed; Lux: light intensity under which the specific scan was performed measured in lux; Std. Dev.: the standard deviation of all the gap distance values measured in mm; Min.: the smallest gap distance value measured in mm; Max.: the largest gap distance value measured in mm; Avg.: the arithmetic mean of all the gap distances measured in mm.

**Table 3:** Data obtained when comparing for precision.

Group	Lux	Mean of *V*	SD of *V*
N.C.	400	0.02039	0.003991
L.N.	1000	0.01714	0.003430
U.J.	3300	0.01662	0.003272
U.J.N.	3800	0.01704	0.002258
U.F.	10000	0.01768	0.002449
U.F.N.	11000	0.02160	0.005800

Group: set of scans conducted under the same simulated conditions; Lux: light intensity under which the specific scan was performed measured in lux; Mean of *V*: the mean of precision values obtained from the metrology software displayed in mm; SD of *V*: standard deviation of the precision values displayed in mm.

## Data Availability

All data is available upon request.
